# Network Risk Diffusion and Resilience in Emerging Stock Markets

**DOI:** 10.3390/e27050533

**Published:** 2025-05-16

**Authors:** Jiang-Cheng Li, Yi-Zhen Xu, Chen Tao

**Affiliations:** 1 School of Finance, Yunnan University of Finance and Economics, Kunming 650221, China; lijiangch@163.com; 2 School of Economics, Yunnan University of Finance and Economics, Kunming 650221, China

**Keywords:** risk diffusion, network resilience, emerging market economies, extreme risk events

## Abstract

With the acceleration of globalization, the connections between emerging market economies are becoming increasingly intricate, making it crucial to understand the mechanisms of risk transmission. This study employs the transfer entropy model to analyze risk diffusion and network resilience across ten emerging market countries. The findings reveal that Brazil, Mexico, and Saudi Arabia are the primary risk exporters, while countries such as India, South Africa, and Indonesia predominantly act as risk receivers. The research highlights the profound impact of major events such as the 2008 global financial crisis and the 2020 COVID-19 pandemic on risk diffusion, with risk diffusion peaking during the pandemic. Additionally, the study underscores the importance of network resilience, suggesting that certain levels of noise and shocks can enhance resilience and improve network stability. While the global economy gradually recovered following the 2008 financial crisis, the post-pandemic recovery has been slower, with external shocks and noise presenting long-term challenges to network resilience. This study emphasizes the importance of understanding network resilience and risk diffusion mechanisms, offering new insights for managing risk transmission in future global economic crises.

## 1. Introduction

In the backdrop of interconnected global financial markets, emerging countries’ stock markets have become key nodes for systemic risk transmission due to their unique market structures and institutional characteristics. Factors like high-frequency trading, cross-border capital flows, and financial derivatives innovation have recently amplified the risk transmission scope, allowing local market shocks to rapidly escalate into systemic crises through channels such as asset price linkages and investor sentiment contagion [1]. This networked risk diffusion pattern transcends traditional financial geography boundaries, necessitating a complex network perspective to re-examine the non-linear interaction mechanisms between financial markets. Studying the network risk diffusion and resilience of emerging countries’ stock markets is crucial for understanding global financial crises, systemic risks, and cross-market risk transmission mechanisms.

The network effect in financial markets creates significant linkages between stock markets in different countries and regions. Market fluctuations can spread through financial networks, destabilizing the entire system. While risk transmission mechanisms are increasingly understood, the resilience of emerging markets to networked risk shocks remains under-researched. For emerging economies, the high correlation in their stock markets makes them more susceptible to external market volatility, especially during substantial global liquidity fluctuations. Compared to developed markets, emerging economies often face issues like insufficient market transparency, lagging regulatory frameworks, and low institutional investor presence. These structural problems make them more vulnerable to external shocks but may also create unique risk buffering mechanisms due to market segmentation [2]. Market resilience refers to the ability to maintain normal operations and recover quickly from external shocks. In emerging stock markets, resilience is typically weak, particularly during global financial crises, capital outflows, or market panics, when these markets experience high volatility and susceptibility to sudden events. Thus, enhancing stock market resilience is essential for mitigating networked risk transmission and ensuring long-term market stability. For instance, Forbes and Warnock [3] found that emerging markets can be both risk sources and “risk buffer zones” due to capital control policies during crises, highlighting the need for dynamic network resilience metrics.

Many traditional financial network models focus mainly on direct transactions or shareholding links between institutions, neglecting the complexity of information flow and risk transmission. Information entropy theory, however, provides a more accurate framework for characterizing financial risks, reflecting the complexity and dynamism of financial markets [4]. Hence, this paper innovatively combines information entropy theory with financial network construction, using transfer entropy to capture nonlinear and directional information flows effectively. Against this context, this study uses information entropy theory to construct a risk association network for the stock markets of 10 emerging economies within the G20, focusing on risk diffusion paths and network resilience evolution. We define cross-border risk spillover directions using risk transfer entropy, analyze risk diffusion with rolling time–window correlations, and identify net risk emitters and recipients. Additionally, we establish a dynamic resilience assessment framework to analyze network resilience changes during normal periods and extreme events (e.g., global financial crises, COVID-19 shocks), revealing resilience variations under external impacts and noise disturbances. Our research not only uncovers cross-border risk diffusion relationships but also provides threshold references for cross-border risk containment policies. The findings will refine monitoring indicators for emerging market systemic risks and offer empirical support for optimizing multilateral financial stability mechanisms.

This paper makes several key contributions: (i) it integrates the risk spillover model from information theory with a dynamic network resilience framework; (ii) it focuses on ten emerging economies within the G20, addressing the gap in previous studies that predominantly focused on developed markets or individual emerging markets without a comprehensive analysis of overall network risk diffusion and resilience; (iii) it applies network resilience methods from biology to the financial domain, proposing the field of financial network resilience research, and examines how network resilience changes under various conditions, including extreme events like the global financial crisis and COVID-19, external shocks, and noise disturbances.

## 2. Related Work

In reality, the interaction between different sectors within the financial market occurs through the transmission of information, and there exists a degree of synchronization between information transmission and risk contagion among these sectors [5]. Hence, it is viable to analyze risk diffusion by quantifying the flow of information among departments. In 2000, Schreiber [6] introduced a method to quantify the entropy of information transmission using information theory [7] and Kullback–Leibler distance. This method, known as transfer entropy (TE), enables the detection of directional information exchange between two systems. Marschinski and Kantz [8] made advancements to Schreiber’s transfer entropy by introducing effective transfer entropy (ETE). Jizba et al. [9] extended transfer entropy to Rényi’s information framework, introducing the concept of Rényi transfer entropy. He and Shang [10] further proposed effective Rényi transfer entropy by combining the principles of effective transfer entropy and Rényi transfer entropy. These developments have garnered significant attention, as transfer entropy is capable of providing clear indications regarding the direction of information flow [5]. In the presence of common risks in the market, the release of negative news can trigger information contagion [11]. The transfer entropy method provides an effective way to measure the directional spillover of information between assets and is relatively straightforward to estimate.

Resilience, as a core concept in complex systems science, has undergone theoretical development through interdisciplinary migration and deepening, from ecology to finance. Holling [12] pioneered the concept of resilience in the study of ecosystem stability, revealing the dynamic mechanisms that maintain system functionality thresholds. Albert et al. [13] proposed a model for the anti-destruction of complex networks, providing a quantitative tool for assessing system vulnerabilities. Dorogovtsev et al. [14] established a model linking system topology and functional stability through complex network theory, laying the theoretical foundation for resilience research. This theoretical framework made groundbreaking progress in the field of financial networks, where Elliott et al. [15] developed an infection model that confirmed the role of network degree distribution in determining risk propagation efficiency. Amini et al. [16] applied percolation theory to discover the quantitative relationship between capital adequacy ratio and the critical point of network phase transition.

The structural characteristics of networks have a dual impact on system resilience. Gai [17] demonstrated that excessive clustering amplifies the diffusion of risks, while Haldane [18] found that moderate clustering can enhance the crisis response capacity of networks. This nonlinear relationship has shifted research focus towards the identification of critical nodes. The DebtRank algorithm proposed by Battiston [19] allows for the dynamic assessment of systemically important nodes, and this methodology was extended by Tobias et al. [20] into a nonlinear risk propagation model based on CoVaR. Recent studies suggest that multilayer network reconstruction strategies can improve financial resilience [21].

Resilience research has made notable progress in interdisciplinary studies. The economic physics approach pioneered by Stanley et al. [22] successfully applied statistical mechanics models to market network simulations. Moro et al. [23] developed a universal framework that enables cross-domain mapping of resilience parameters between ecological systems and financial networks. Currently, resilience research is focusing on machine learning-based high-dimensional network resilience prediction [24], dynamic network phase transition control theory [25], and enhancing the predictive accuracy of resilience models [26]. Researchers have also developed dynamic network approaches based on information entropy to assess the resilience state of networks [27]. These advances support the macroprudential regulatory framework for financial stability.

It can be observed that traditional risk contagion models often rely on multivariate GARCH and Copula techniques [28], with less focus on information transmission. Transfer entropy, which quantifies uncertainty in information flow, has an advantage over Granger causality due to its superior handling of nonlinear relationships [29], providing deeper insights into market risk transmission. Additionally, network state prediction is key to resilience assessment. Methods using information entropy focus more on information flow, enhancing efficiency [30]. This paper differs by exploring how major events impact risk network structures, effectively illustrating temporal contagion attributes, and evaluating network resilience across different time states using information entropy.

The network risk diffusion and network resilience of emerging market economies remain areas that require further exploration. Previous studies have used complex networks to model financial risk contagion [31], quantifying uncertainty and diversity with information entropy [32] and measuring resilience through network robustness [33]. This study innovatively integrates network theory with transfer entropy to define risk contagion paths in emerging markets from the perspective of information asymmetry. It combines information entropy and network resilience to quantify the evolution of financial network states under high uncertainty. Building on existing research, this study integrates the transfer entropy framework from information systems and the resilience theory from ecological systems. We focus on the 10 emerging economies within the G20, as classified by the International Monetary Fund (IMF), to investigate the risk diffusion and resilience of their stock market networks. By measuring risk transfer entropy, we define the direction of cross-border risk spillovers, and reveal the changes in network resilience under various conditions, including the original network, extreme event windows (such as the global financial crisis and the COVID-19 pandemic impact), external shocks, and noise disturbances. Based on the research objectives and structure, this paper is organized as follows: Section 3 introduces the transfer entropy method and the network resilience measurement approach adopted in this study, while Section 4 presents a summary and analysis of the empirical results. Finally, Section 5 concludes with the main findings of the study.

## 3. Methodology

### 3.1. Transfer Entropy and Effective Transfer Entropy

In the analysis of complex systems, quantifying the flow of information between variables is crucial. Transfer entropy (TE), a concept introduced by Schreiber [6], has become an effective tool for measuring the nonlinear Granger causality between two systems. Its theoretical foundation is rooted in Shannon entropy from information theory, which provides a quantitative method for evaluating the amount of information by measuring the uncertainty in data. Specifically, for a discrete random variable *X* with probability distribution $p(x_{t})$, Shannon entropy $H_{X}$ is defined as the weighted average of all possible values $x_{t}$, expressed as:

(1) $$H_{X}=-\sum_{x_{t}}^{N}p\left(x_{t}\right)logp\left(x_{t}\right).$$

The joint probability p(x,y) is essential for describing systems involving two independent variables *X* and *Y*, as well as their interactions. The corresponding joint entropy $H_{X,Y}$ is expressed as:

(2) $$H_{X,Y}=\sum_{x\in X}\sum_{y\in Y}p\left(x,y\right)logp\left(x,y\right).$$

Furthermore, utilizing the framework of generalized Markov processes, transfer entropy from process *Y* to process *X* is derived, with the following formula for calculation:

(3) $$TE_{X\leftarrow Y}\left(k,l\right)=\sum p\left(x_{t+1},x_{t}^{\left(k\right)},y_{t}^{\left(l\right)}\right)log\frac{p(x_{t+1}|x_{t}^{\left(k\right)},y_{t}^{\left(l\right)})}{p(x_{t+1}|x_{t}^{\left(k\right)})}.$$

The indicator $TE_{X\leftarrow Y}$ directly reflects the strength of information transfer from *Y* to *X*. A positive value implies that $x_{t+1}$ is significantly influenced by *Y*, and the greater the TE value, the stronger the influence.

However, in practical applications, calculating TE faces several challenges, particularly due to non-smooth data noise and finite sample effects, which may introduce bias. This bias can be mitigated by computing the Effective Transfer Entropy (ETE) [5]. The core idea is to first randomize all process components, thus disrupting the original Granger causality while maintaining the probability distribution within each process. This results in the calculation of Random Transfer Entropy (RTE). Then, by subtracting RTE from the original TE, the ETE can be obtained, which removes the noise interference. Its mathematical expression is:

(4) $$ETE_{X\leftarrow Y}\left(k,l\right)=TE_{X\leftarrow Y}\left(k,l\right)-RandomTE_{X\leftarrow Y},$$

where $RandomTE_{X\leftarrow Y}$ represents the random transfer entropy, which is the average entropy value computed after randomizing the variables $y_{t}$ and $x_{t}$, expressed as:

(5) $$RandomTE_{X\leftarrow Y}=\frac{1}{M}\sum_{t=1}^{M}TE_{Y_{shuffled}\to X^{\left(k,l\right)}},$$

where *M* denotes the total time length. When the ETE value is negative, it indicates that the estimated expected effective transfer entropy exceeds the actual effective transfer entropy, and as the ETE value approaches zero, this difference gradually diminishes. According to the study [8], in our research, we select the number of shuffles to be 50 and the bootstrap sample size to be 100.

At the system level, to comprehensively assess the overall information flow among the 10 emerging countries in the G20 group, we draw upon the methodology proposed by Ji et al. [11] and integrate the connectivity framework established by Diebold and Yilmaz [2]. This study constructs a transfer entropy matrix. By calculating the total transfer entropy between all pairs of variables, the overall system transfer entropy is obtained, with the following formula:

(6) $$\;T_{total}=100*\frac{1}{N}\sum_{i,j=1}^{N}T_{ij},i\neq j.$$

where $T_{ij}$ represents the transfer entropy value between variables *i* and *j*, and *N* is the total number of variables in the system. The overall transfer entropy provides a macro perspective for quantifying the level of information interaction across the entire system.

By subtracting the random baseline to actively eliminate finite sample bias [6,8], this approach not only captures direct risk transmission between stock markets but also reveals potential risk propagation pathways triggered by information flow. This method addresses the limitations of traditional networks based solely on transactional relationships, which may overlook implicit connections and complex transmission channels. Furthermore, embedding these directional indicators into a rolling window topology allows for the observation of the temporal evolution of system relationships, thereby enhancing early warning capabilities.

### 3.2. Network Resilience

Risk diffusion serves as the foundation for studies on network resilience. Existing research has demonstrated the impact of node-based diffusion on network resilience [34], while networks with high resilience may also reduce the spread of risks in uncertain environments [35]. In the field of biological protein research, the relationships and interactions between networks are crucial, with network resilience depending on its ability to withstand external shocks and noise disturbances [36]. Inspired by this concept, we transfer the computational methods for network resilience from biology to the financial domain, making appropriate adjustments to adapt them for measuring the resilience of networks formed between financial systems. By iteratively adding and removing nodes in the network, we measure network resilience indicators under different scenarios to infer the stability characteristics of the financial network.

Information entropy effectively quantifies the contribution of nodes to the overall activity of a network, capturing the global state of high-dimensional complex systems more accurately than traditional methods [27]. In addition to reflecting the dynamic changes of the network in response to disturbances, information entropy can also serve as an optimization objective to guide the system’s recovery process, thereby enhancing network resilience more effectively [30]. For the network G(V,E), where *V* is a set of *N* nodes and *E* is the set of edges, we construct the network among the 10 emerging countries in the G20 group by calculating the correlation matrix. Let the total number of node connections in the network be *M*. Using information entropy as a quantitative measure of network resilience, we define the calculation of network resilience. The information entropy is measured as:

(7) $$\;H\left(G_{f}\right)=-\frac{1}{\log(N)}\sum_{x=1}^{X}\varphi_{x}\log\varphi_{x},\;$$

where $c_{x}$ represents the connection between two adjacent nodes. The probability of any node having $\varphi_{x}$ comes from $c_{x}$, and $\varphi_{x}=\left|c_{x}\right|/N$, satisfying $\sum_{x=1}^{X}c_{x}=N$.

Further, removing a node is considered equivalent to removing all of its connections, turning it into an isolated node. The proportion of nodes removed relative to the total number of nodes in the network is denoted as *f*. We consider removing a proportion *f* (*f* = 0 to 1) of the nodes in the network. For each value of *f*, we perform 20 simulation runs, each time removing 0%, 1%, …, 100% of the nodes. The information entropy after node removal at each proportion is calculated, and the integral area under the entropy curve after each iteration is computed to quantify the network resilience R(G), which is calculated as:

(8) $$R\left(G\right)=1-\sum_{f=0}^{1}\frac{H(G_{f})}{r_{f}},\;$$

where $r_{f}$ represents the removal rate of nodes. In this study, we set $r_{f}$ to 100 by default.

In real financial systems, node removal signifies the exit of key participants. For example, a financial institution’s bankruptcy removes its node, while trade blockades can expel countries from the trade network. Node removal severs connections to linked nodes, potentially undermining the network’s stability and resilience.

We systematically analyze the variation in network resilience under three different scenarios: First, the resilience of the original network, which intuitively reflects the network’s ability to resist shocks in its normal state. Second, the network resilience under extreme shocks, examining the stability of the network, which is already in an extreme event state, when facing a second major unexpected shock. Third, the network resilience under noise disturbances, exploring the performance of the network under the influence of random disturbances. By comparing the network resilience across these three scenarios, we gain a comprehensive understanding of the stability and vulnerability characteristics of the financial network.

## 4. Results

### 4.1. Data

This study examines 10 emerging markets from the IMF’s G20 list, as their economic and financial scale reflect broader emerging market trends. Developed markets like the US, EU, and Japan are excluded due to their spillover effects, which could distort the risk diffusion mechanisms within emerging markets. G20 markets offer more complete data, whereas smaller markets like Vietnam and Nigeria have limited, inconsistent data.

The characteristics of stock index data, such as insufficient liquidity and asynchronous timeframes, may impact the accuracy of transfer entropy calculations [5,8]. To mitigate these effects, methods such as data cleaning, interpolation, standardization, and model adjustments [8] are employed, followed by robustness testing to ensure the reliability of the results.

According to the classification by the IMF, our study focuses on emerging economies within the G20 group, including China, India, Brazil, Russia, South Africa, Indonesia, Argentina, Mexico, Turkey, and Saudi Arabia, analyzing the relevant data for these countries. The selection of stock indices is provided in the Table A1. All data were obtained from the Wind database.

**Figure 1:**
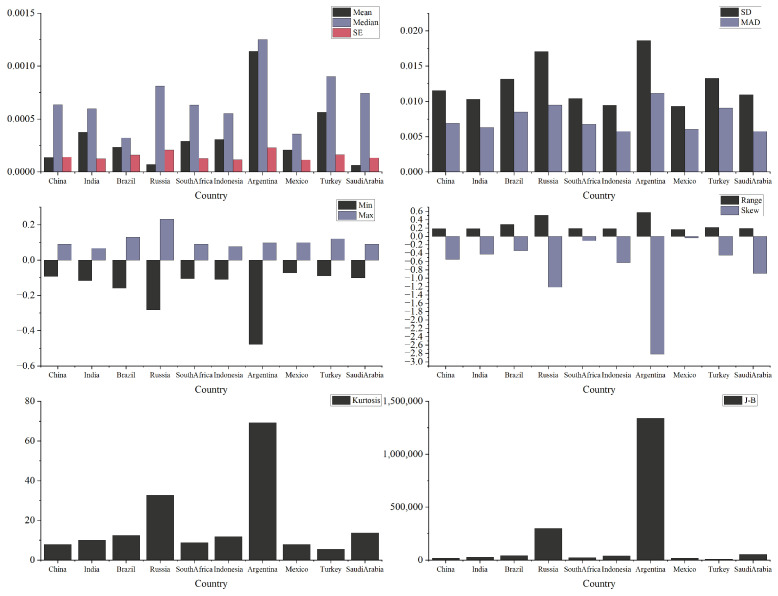
Descriptive statistical results.

Using return data from 6672 trading days across these 10 countries, we derived the descriptive statistics presented in Figure 1. The results clearly illustrate the overall characteristics of the sample. In terms of mean values, most countries’ data are concentrated around similar averages, with Argentina exhibiting the highest mean and Russia the lowest, indicating a variance in overall levels. Argentina’s data shows the greatest standard deviation (SD), indicating significant volatility and notable differences between individual data points, suggesting that crisis events may have a persistent impact on its volatility structure. This is further supported by the Median Absolute Deviation (MAD). Regarding skewness, all countries’ data exhibit left-skewed distributions, with negative skewness values. In terms of kurtosis, all countries show values greater than 3, indicating that the data distributions are more peaked than normal distributions, with “leptokurtic” tails. This phenomenon aligns with the characteristics of emerging market vulnerabilities. Notably, Argentina shows the highest kurtosis, indicating a highly concentrated data distribution and more pronounced tail risks. The P-values from both the Augmented Dickey–Fuller (ADF) test and Jarque–Bera (JB) test indicate that the null hypothesis of normality is rejected for all countries, suggesting that the data exhibit non-normal features, such as asymmetry and heavy tails.

### 4.2. Analysis of Risk Transfer Entropy in Emerging Market Countries

Utilizing the transfer entropy model, we calculated the risk interconnections among ten emerging market countries, with the results presented in Table 1 and Table 2. Table 1 illustrates the pairwise transfer entropy between the ten countries, while Table 2 highlights the primary influencing markets and the markets being influenced for each country. Notably, Brazil’s transfer entropy to India, Mexico’s transfer entropy to India, and Mexico’s transfer entropy to Indonesia are relatively high, at 0.0165, 0.0165, and 0.0169, respectively. These values suggest considerable risk transmission between these countries.

Several factors could contribute to these outcomes. First, as a major global exporter of resources, Brazil’s economic fluctuations affect India through commodity trade channels. Additionally, Mexico transmits risks to India and Indonesia via its manufacturing exports and global supply chain connections. On the other hand, the transfer entropy values between China and Argentina, Argentina and Mexico, as well as Saudi Arabia and Argentina, are extremely low, indicating minimal risk transmission. This may be attributed to capital controls and market segmentation, which reduce international capital flows and market interconnectedness [37], thus diminishing the transmission of external shocks. Additionally, differences in economic structures and financial markets further weaken the spillover effects of risks [38].

To further explore the risk-exporting and risk-receiving countries among the 10 countries, we examined the FROM, TO, and NET values in the transfer entropy analysis. The detailed results are presented in Table 3. As shown in Table 3, from the perspective of risk export, Brazil, Mexico, and Saudi Arabia are the primary risk-exporting countries, mainly due to their roles as commodity and energy exporters. Brazil has the highest TO value of 0.0817, indicating its strong risk-exporting capacity. This can be attributed to its position as a major global exporter of agricultural and mineral products [39]. Mexico also exhibits significant risk transmission, which is largely tied to its close economic relations with the United States. As a member of the North American Free Trade Agreement (NAFTA), Mexico’s economy heavily relies on exports to the U.S. Changes in U.S. economic policies, the rise of trade protectionism, and economic fluctuations in North America can lead to risk spillovers through trade and investment channels, further affecting other countries. Saudi Arabia follows with a notable risk-exporting ability, mainly stemming from its position as one of the world’s largest oil exporters. Fluctuations in oil prices not only affect its fiscal revenues but also trigger cascading effects on other countries through the global energy supply chain.

On the other hand, from the perspective of risk input, India, South Africa, Indonesia, and Russia exhibit higher risk-receiving characteristics [40]. India has the highest FROM value, indicating its strong capacity to absorb risks. This is related to its financial market openness and the uncertainty surrounding its economic policies. Fluctuations in the Indian stock market may influence other emerging markets, especially those with close investment ties, such as South Africa and Indonesia. Additionally, India’s economic reforms and adjustments in monetary policy could create ripple effects in the region, impacting the economic environment of neighboring countries. South Africa’s FROM value ranks second, with its risk absorption linked to its position as a major African economy and its close ties with global markets. Its relatively open financial markets make it susceptible to international capital flows and global market volatility. Indonesia’s risk input is primarily associated with its position as the largest economy in Southeast Asia and its connections to global markets. The country’s economic structure, heavily reliant on resource exports and manufacturing, makes it vulnerable to external risks due to fluctuations in global demand and prices for these products. Russia’s risk input is predominantly linked to its dominant position in oil and gas exports. The dual pressures of international oil price fluctuations and geopolitical risks make Russia susceptible to external risks. A drop in oil prices can reduce Russia’s fiscal revenues and weaken its economic strength, while geopolitical tensions may lead to capital outflows and financial market instability, thus affecting domestic economic stability.

China, Turkey, and Argentina have relatively low TO, FROM, and NET values, indicating their limited influence in the emerging market economies [40]. China’s NET value is negative, suggesting that its risk input slightly exceeds its risk output. The findings of this study are consistent with those of Patra and Panda (2021) [37], which could be attributed to China’s economic policies and foreign capital controls [41]. Given China’s economic scale and global influence, it plays a complex role in risk transmission. As the world’s manufacturing hub, China’s economic policy adjustments, market demand fluctuations, and supply chain disruptions can transmit risks to other countries through trade and investment channels. However, China is also actively implementing measures to strengthen financial regulation and economic restructuring to mitigate the impact of external risks. Turkey’s NET value is also negative, with its risk input slightly surpassing its risk output. The risk correlation in Turkey is influenced by its geopolitical position and the degree of its economic openness. Argentina’s NET value is positive, indicating that its risk output is slightly higher than its risk input. Argentina’s economic structure is heavily dependent on agricultural and livestock exports, and fluctuations in global food market demand and price volatility significantly affect its risk situation. For instance, a global food crisis or major fluctuations in food prices could directly impact Argentina’s export revenue and economic growth, thereby transmitting risks to other countries through trade channels.

**Figure 2:**
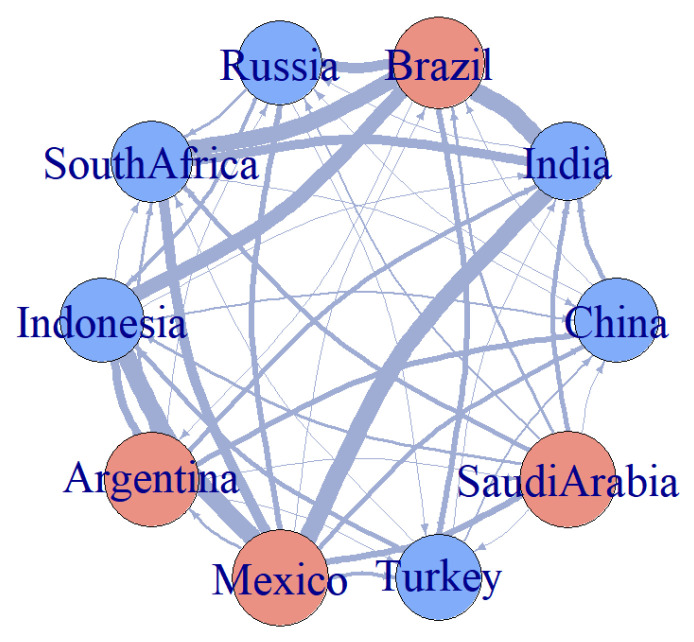
Risk Network of Emerging Market Countries.

We construct a risk association network for emerging market countries based on the net transfer entropy between pairs of markets, further illustrating the input and output relationships of risk among the ten emerging market countries. In the network, red nodes represent risk-exporting countries, while blue nodes signify risk-inputting countries. The size of the nodes in the network is determined by their degree, which reflects the centrality of each country in risk transmission. Figure 2 presents the risk association network, showing that the results align with those of the previously analyzed net transfer entropy. Argentina, Mexico, Brazil, and Saudi Arabia emerge as the main risk-exporting countries, while the remaining countries are identified as risk-inputting countries. We compute transfer entropy values with varying parameters, and the results across time windows are consistent (see Table A2), ensuring model stability and providing strong support for studying risk transmission in emerging markets.

### 4.3. Analysis of the Risk Correlation Among Emerging Market Countries

**Figure 3:**
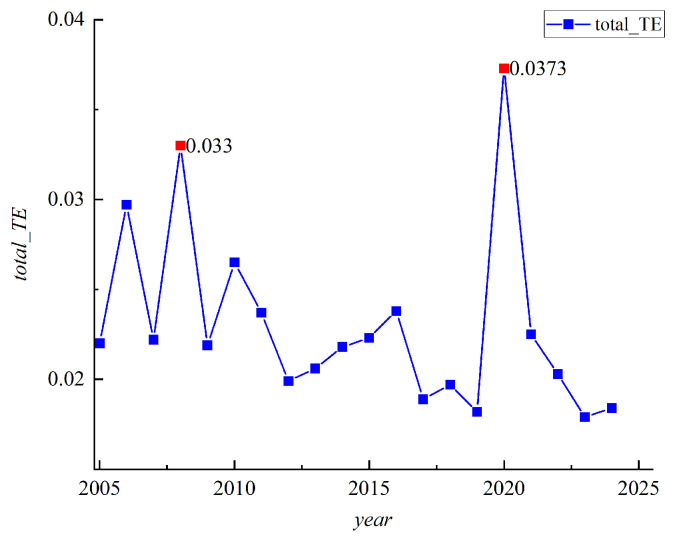
Risk Correlation among Emerging Market Countriess.

To further assess the overall risk correlation level among emerging market countries, we calculate the total transfer entropy based on the Diebold–Yilmaz (DY) spillover framework. A higher value of total transfer entropy indicates stronger overall risk correlation between markets and more significant spillover effects of risk across markets. Additionally, we introduce a rolling time window in our study. From 2005 to 2013, the average number of trading days per year was 360, while from 2014 to 2024, it was 310. Therefore, the corresponding rolling window values are selected for analysis during different study periods. The results are shown in Figure 3.

Upon observing the results in Figure 3, it is evident that the overall risk correlation level between markets exhibits distinct volatility characteristics. During the 2008 global financial crisis, the total transfer entropy reached a peak, reflecting the rapid transmission and diffusion of risk across markets. From 2010 to 2012, markets gradually stabilized, leading to a decline in the total transfer entropy value. Between 2016 and 2018, the total transfer entropy fluctuated upwards, likely due to events such as the Brexit referendum and the US tax reduction policy. During the 2020–2021 COVID-19 pandemic, the total transfer entropy reached its highest value over the entire sample period, significantly enhancing market correlations and causing rapid risk transmission. Although the total transfer entropy decreased between 2022 and 2024, it remained at a relatively high level.

It is evident that the overall risk correlation level among emerging market countries experienced significant peaks in 2008 and 2020. The notable peak observed around 2008 coincides with the occurrence of the global financial crisis. During the crisis, global financial markets were turbulent, and risks rapidly spread across various markets. In particular, the financial turmoil caused by the US subprime mortgage crisis quickly affected global stock, bond, and foreign exchange markets, leading to a substantial increase in market correlation, and significantly amplifying risk spillover effects. As a result, the total transfer entropy surged to a high level. In the 2020–2021 period, the total transfer entropy reached its highest value of the entire sample period, coinciding with the crisis triggered by the COVID-19 pandemic. The public health crisis and economic shutdown induced by the pandemic caused significant disruptions to global financial markets, with sharp fluctuations in stock, bond, and commodity markets. The correlation between markets substantially strengthened under the impact of the pandemic, leading to rapid risk propagation and a sharp rise in the total transfer entropy.

### 4.4. Analysis of the Network Resilience Among Emerging Market Countries

**Figure 4:**
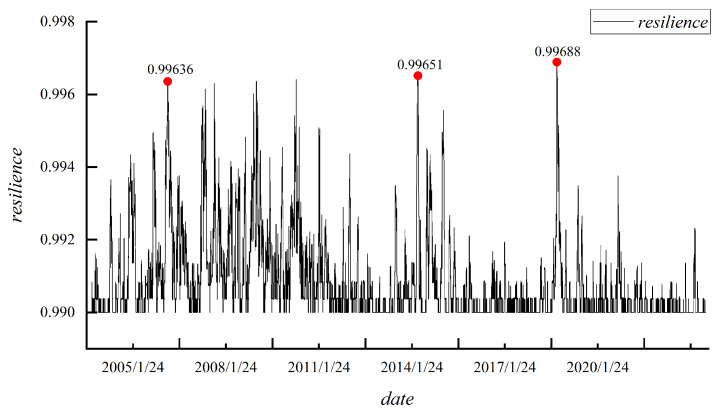
Network Resilience among Emerging Market Countriess.

Network resilience refers to a network’s ability to respond to and recover from external shocks or extreme events. Figure 4 illustrates the changes in the correlation network resilience of emerging countries. Upon examining the results shown in the Figure 4, it is evident that resilience exhibits notable fluctuations. The peaks in resilience are observed in 2007, 2015, and 2020. Prior to 2014, the fluctuations in resilience were more pronounced, whereas after 2014, resilience changes were more stable. Specifically, before the 2008 financial crisis, the economies and financial systems of emerging market countries were relatively independent, with lower levels of globalization. After the crisis broke out, the correlation among emerging market countries surged rapidly, reflecting the global economic network’s shock due to the financial crisis, which led to a temporary increase in correlation network resilience. This may have been a key factor contributing to the peak in resilience observed in 2007. After the financial crisis, various stimulus policies were implemented globally, leading to a gradual recovery of the global economy. However, due to the differing policies each country adopted to cope with the crisis, the recovery paths of emerging market countries varied, resulting in substantial fluctuations in resilience. Consequently, before 2014, resilience fluctuated more significantly due to instability in global capital flows, uncertainty in international market demand, and structural adjustments in various economies, which further increased the volatility of emerging market countries’ correlation networks.

After 2014, with the economic stabilization and recovery post-financial crisis, many countries reduced external risks to their economic systems by strengthening macroprudential policies, establishing monetary policy frameworks, and coordinating policies. Additionally, the prices of global commodities, especially energy, metals, and agricultural products, exhibited significant fluctuations, which directly impacted resource-exporting emerging market countries. This led to structural adjustment pressures in many resource-dependent economies. This adjustment process was relatively smooth, resulting in more stable changes in resilience among emerging market countries. The outbreak of the COVID-19 pandemic in 2020 severely impacted the global economic system, particularly in emerging market countries. In the early stages of the pandemic, global economic activity almost came to a halt, and global supply chains were severely disrupted, leading to a peak in resilience in 2020. This peak reflects the close correlations among emerging market countries. Post-pandemic, the asynchronous global economic recovery also led to fluctuations in the correlation network.

Based on the above analysis, the risk correlation levels of emerging market countries reached notable peaks in 2008 and 2020, and the network resilience also exhibited clear peaks in 2007 and 2020. Using transfer entropy and network resilience, we identified two critical nodes: the 2008 financial mortgage crisis and the 2020 COVID-19 pandemic as the extreme event windows. By dividing the timeline, we constructed the pre-event, during-event, and post-event correlation networks of emerging market countries. The specific time divisions are presented in Table 4.

**Figure 5:**
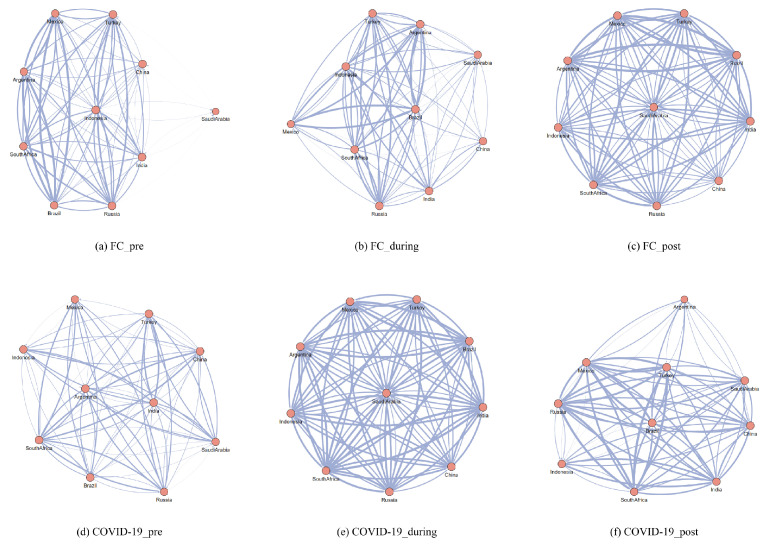
Correlation Network of Extreme Events in Emerging Market Countries. The red nodes represent different emerging market countries, while the blue lines indicate the relationships between these countries. The thicker the line, the stronger the connection between them.

Based on the time periods established, we constructed the pre-event, during-event, and post-event correlation networks. As shown in Figure 5, panels (a)–(g) represent the emerging market countries’ correlation networks before, during, and after the financial crisis and COVID-19 pandemic, respectively. As in previous descriptions, the nodes in the network represent emerging market countries, and the connections between nodes indicate correlations between countries. The thickness of the connections represents the strength of the correlation; the thicker the connection, the greater the correlation.

Figure 5a–c demonstrates that prior to the 2008 financial crisis, emerging market countries did not show strong correlations. During the crisis, the correlation weakened, but it increased after the crisis. A possible explanation for this change is that before the financial crisis, the global economy was in a relatively stable growth phase, with limited connections between the emerging market economies. Their dependency on market demand and external capital inflows was more significant, which led to weaker inter-country correlations [48,49]. When the crisis occurred, the global financial system experienced a large-scale shock, and the capital mobility of emerging market countries was relatively low, with delayed responses to external shocks. As a result, their correlations did not increase, but rather decreased due to different economic performances and market fluctuations across countries [38]. After the crisis, as the global economy began to recover, the economic growth rate of emerging market countries, particularly China and India, started to pick up. Capital flowed back into emerging markets, leading to closer economic and financial ties between countries. Simultaneously, the deepening of global supply chains and financial networks contributed to the strengthening of correlations between countries.

Figure 5d–f show the changes in correlations among emerging market countries before, during, and after the COVID-19 pandemic that began at the end of 2019. It can be observed that before the pandemic, the economies of emerging market countries were relatively dispersed, and there were significant differences in their industrial structures and stages of economic development. Their dependencies on demand, capital, and resources varied. Despite the acceleration of globalization, their economic connections remained weak. Particularly, after the pandemic outbreak, the global economy faced unprecedented shocks, leading to drastic global fluctuations. The correlations between countries actually strengthened [50]. This result is consistent with the earlier analysis of resilience changes. Economic crises transmitted between countries, and financial links exhibited stronger correlations. Increased communication, cooperation, and mutual support among countries further raised the correlations. As the pandemic was gradually controlled and the global economy began to recover [51], the spread of the crisis was managed, and the economic and financial ties between countries gradually returned to normal levels.

**Figure 6:**
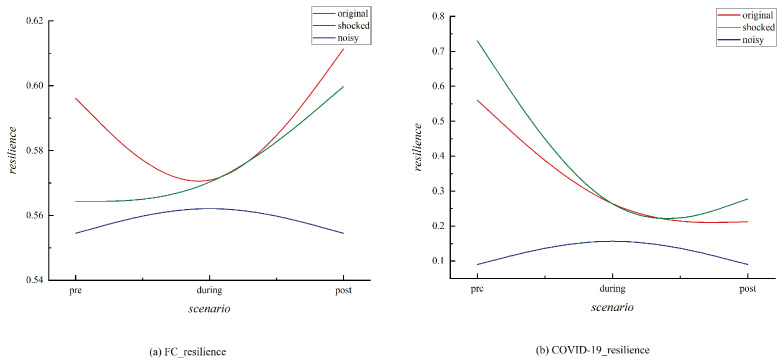
Resilience of the Correlation Network of Extreme Events in Emerging Market Countries Under Different Scenarios. The vertical axis in the figure represents network resilience, while the horizontal axis denotes various periods of extreme events. The red curve represents the original network, the green curve illustrates the network subjected to subsequent extreme shocks, and the blue curve depicts the network affected by random fluctuations and noise disturbances.

To further explore the evolution of resilience in emerging market countries’ networks during extreme events, we calculated the network resilience for different scenarios. We considered three different situations: the original network, the network exposed to extreme shocks again, and the network affected by random fluctuations or noise disturbances. We investigated the network resilience under these three conditions, as shown in Figure 6, which presents the resilience of the emerging market countries’ extreme event correlation networks.

For the original network, when extreme events occur, resilience weakens, and instability increases. After the 2008 financial crisis, resilience gradually rebounded, while following the COVID-19 pandemic in 2020, although there was a recovery, the pace was slower. Extreme events, such as the 2008 financial crisis and the 2020 pandemic, have a profound impact on the stability of networks. Following the 2008 crisis, the resilience of the original network gradually recovered, which could be attributed to market adjustments and policy interventions after the crisis. Many new policies helped rebuild the economy and financial system, thereby improving network resilience [52]. However, after the COVID-19 pandemic in 2020, although resilience gradually recovered, the speed of recovery was slower. This could be due to the far-reaching effects of the pandemic, particularly the long-term changes it induced in global supply chains, labor markets, and consumption patterns, which made the network’s recovery process more complex and prolonged [53]. Moreover, the global scale and uncertainty of the pandemic increased the difficulty of recovery for many countries, with economic recovery being slower than the one following the 2008 financial crisis.

For networks subjected to repeated extreme shocks, the resilience of the network in 2008 increased when a certain degree of extreme shock was applied during the crisis period. This phenomenon aligns with the findings of Charmantier et al. [54], which suggest that “adversity promotes adaptation”. In other words, environmental changes drive the enhancement of biological populations’ adaptability, and similarly, in times of crisis, they can stimulate the reconfiguration and innovation of network resources, thereby improving the adaptability of the network. Many businesses underwent restructuring after the crisis, eliminating inefficient components and strengthening the resilience and efficiency of the network [55]. This aligns with the “self-organization” feature in systems theory, which suggests that when systems are under extreme pressure, parts of the network may spontaneously adapt and evolve, thereby enhancing the overall resilience of the system in the long term. During the 2008 crisis, network resilience improved, but during the 2020 COVID-19 pandemic, the external extreme shock caused a sharp decline in network resilience. The impact of the pandemic on economic activity was more widespread and abrupt than the 2008 financial crisis. This unpredictability and long-lasting nature increased the complexity of network recovery [56]. Specifically, if moderate shocks are applied in advance, they can stimulate the network’s adaptability, strengthen its resistance to future shocks, and enhance its resilience [57].

Regarding networks subjected to noise disturbances, both periods exhibited the same trend: after applying a certain degree of noise, resilience improved, but the increase was not significant. Noise disturbances are usually external disruptions, which may enhance the system’s adaptability in the short term. However, due to the randomness and uncontrollability of disturbances, their long-term impact on the system is unstable, and they are likely to weaken the system’s adaptability. In network resilience, applying some noise disturbance can trigger temporary adaptive responses within the network, but these responses are unlikely to last and may introduce excessive complexity, thereby reducing resilience in the long run [58]. Moderate noise or disturbances can enhance system resilience. Therefore, it is beneficial to introduce a wider range of diverse and unforeseen noise scenarios to more comprehensively assess the resilience of the financial system, rather than solely relying on standardized shocks. By simulating the response of the financial system to moderate noise, policy implementation can better align with the genuine needs of system resilience [18]. Alternatively, regulatory sandboxes can be designed as controlled experimental mechanisms to strengthen the resilience of the financial system [59].

In addition, the results in Figure 6 show that the changes in network resilience during 2008 and 2020 reflect the influence of extreme events on system adaptability and recovery capacity. The 2008 financial crisis enhanced network resilience through external shocks and self-organization mechanisms, while the impact of the 2020 COVID-19 pandemic, due to its global and complex nature, led to a slower recovery process. In contrast to the liquidity issues that characterized the 2008 financial crisis, the pandemic inflicted severe damage on the real economy, triggering disruptions in supply chains and deeply impairing economic activities. This resulted in a slower and more complex recovery process for the financial system. Consequently, the restoration of financial network resilience following the pandemic has been more gradual compared to the aftermath of the 2008 crisis [60]. While noise disturbances can improve resilience in the short term, excessive disturbances may weaken the system’s overall stability in the long run [61].

## 5. Discussion and Conclusions

As globalization deepens, economic ties between emerging market economies have become increasingly interconnected. Understanding the mechanisms of risk transmission among these nations is essential for developing more effective risk management strategies. Furthermore, investigating the evolution of network resilience and its characteristics can help enhance the stability of the global economic system. This study, based on the transfer entropy model, analyzes the risk diffusion and interconnections among ten emerging market countries and explores the risk output and input characteristics of each nation. The findings reveal that Brazil, Mexico, and Saudi Arabia are the primary risk-exporting countries, with their risk transmission capabilities primarily linked to commodities and energy. Notably, Brazil exhibits the strongest risk export capacity, which is closely tied to its position as a major global exporter of agricultural and mineral products. Mexico’s risk diffusion mainly occurs through its strong economic ties with the United States, while Saudi Arabia impacts other countries via its oil exports. Conversely, India, South Africa, Indonesia, and Russia demonstrate significant risk reception capacities, which are closely associated with their open financial markets, dependence on resource exports, and geopolitical factors. In particular, India’s financial market uncertainty makes it a key risk inputter. Through the construction of a risk association network, the study further confirms that Brazil, Mexico, and Saudi Arabia are the primary risk exporters, while other countries predominantly assume the role of risk inputters.

Moreover, the research incorporates the DY total risk spillover framework and a rolling time-window approach to dynamically analyze the overall risk interconnection levels among markets. The results indicate that the global financial crisis of 2008 and the COVID-19 pandemic in 2020 were critical events that caused significant fluctuations in the risk association levels of emerging market economies. During these extreme events, risk transmission and diffusion were greatly intensified, particularly during the 2020 pandemic, when the total transfer entropy reached its highest value of the study period, highlighting the close linkages and risk diffusion effects within the global economy and financial markets. Additionally, network resilience analysis shows that a certain level of noise contributes to enhancing network resilience. Following the financial crisis, the global economy and financial system gradually recovered, with macroeconomic policy adjustments strengthening the stability of the network. However, the post-pandemic recovery process has been slower, demonstrating the complex impact of long-term changes in global supply chains, labor markets, and consumption patterns on network resilience.

By systematically evaluating the risk diffusion mechanisms and changes in network resilience among emerging market economies through the transfer entropy model and network resilience analysis framework, this study reveals the profound impact of globalization and extreme events on the risk interconnections of emerging market nations. The analysis of events such as the 2008 financial crisis and the 2020 COVID-19 pandemic provides a new perspective on risk transmission and offers policy recommendations for addressing risk propagation in future global economic crises.

Based on the findings, we propose several policy measures: First, regulators should identify key risk-exporting and receiving countries and enhance supervision, such as focusing on commodity and energy export-related financial activities in countries like Brazil, Mexico, and Saudi Arabia to prevent risk spillover. Second, policymakers should adjust cross-border capital flow and macroprudential policies according to the dynamic changes in the risk correlation network, especially during or after extreme events like the global financial crisis, to stabilize markets and reduce risk diffusion. For instance, during the 2008 crisis, tightening controls on short-term speculative capital flows could have mitigated financial instability. Additionally, monitoring changes in network resilience aids financial stability management. Controlled disturbances or regulatory sandboxes can more accurately assess resilience, aligning with real financial needs.

This study offers a fresh perspective on risk contagion and enhances the literature on risk correlation networks. However, it focuses on G20 emerging markets, leaving contagion in developed and non-G20 emerging markets unexamined. Additionally, daily data may miss intra-day fluctuations, and the bias correction of transfer entropy depends on lag selection. Future work could build multilayer networks, use high-frequency data, and apply machine learning to improve resilience forecasting.

## Figures and Tables

**Table 1:** Transfer Entropy Between Emerging Market Countries.

Country	China	India	Brazil	Russia	South Africa	Indonesia	Argentina	Mexico	Turkey	Saudi Arabia
China	0	0.0062	0.0045	0.0040	0.0035	0.0011	0.0000	0.0021	0.0025	0.0004
India	0.0023	0	0.0041	0.0044	0.0037	0.0046	0.0019	0.0036	0.0032	0.0015
Brazil	0.0038	0.0165	0	0.0130	0.0164	0.0156	0.0025	0.0016	0.0076	0.0047
Russia	0.0030	0.0035	0.0051	0	0.0031	0.0064	0.0020	0.0037	0.0011	0.0032
South Africa	0.0039	0.0106	0.0052	0.0009	0	0.0049	0.0023	0.0061	0.0014	0.0012
Indonesia	0.0029	0.0061	0.0047	0.0033	0.0052	0	0.0019	0.0038	0.0033	0.0009
Argentina	0.0044	0.0055	0.0022	0.0030	0.0043	0.0083	0	0.0004	0.0028	0.0011
Mexico	0.0059	0.0165	0.0018	0.0079	0.0135	0.0169	0.0026	0	0.0066	0.0039
Turkey	0.0040	0.0037	0.0037	0.0004	0.0018	0.0070	0.0014	0.0035	0	0.0024
Saudi Arabia	0.0020	0.0049	0.0072	0.0049	0.0051	0.0032	0.0002	0.0094	0.0038	0

**Table 2:** The primary driving factors of risk export or input in emerging markets.

Country	Export Risk Driver	Input Risk Driver
China	India (0.0062)	Mexico (0.0059)
India	Indonesia (0.0046)	Mexico (0.0165), Brazil (0.0165)
Brazil	India (0.0165)	Saudi Arabia (0.0072)
Russia	Indonesia (0.0064)	Brazil (0.0130)
South Africa	India (0.0106)	Brazil (0.0164)
Indonesia	India (0.0061)	Mexico (0.0169)
Argentina	Indonesia (0.0083)	Mexico (0.0026)
Mexico	Indonesia (0.0169)	Saudi Arabia (0.0094)
Turkey	Indonesia (0.0070)	Brazil (0.0076)
Saudi Arabia	Mexico (0.0094)	Brazil (0.0047)

**Table 3:** Net Transfer Entropy among Emerging Market Countries.

Variable	TO	FROM	NET
China	0.0243	0.0322	−0.0079
India	0.0293	0.0735	−0.0442
Brazil	0.0817	0.0383	0.0433
Russia	0.0311	0.0419	−0.0107
South Africa	0.0365	0.0567	−0.0202
Indonesia	0.0321	0.0681	−0.0359
Argentina	0.0320	0.0148	0.0173
Mexico	0.0754	0.0342	0.0412
Turkey	0.0279	0.0323	−0.0044
Saudi Arabia	0.0408	0.0192	0.0216

**Table 4:** Division of Extreme Event Time.

Event	Time	Days	Reference
2008 Financial Crisis	1 July 2006–9 August 2007	769 (Risk Build-up)	Brunnermeier (2009) [42]
	10 August 2007–15 September 2008	402 (Outbreak of Crisis)	Gorton & Metrick (2012) [43]
	16 September 2008–9 March 2009	175 (Policy Response)	Taylor & Williams (2009) [44]
COVID-19	1 December 2019–23 January 2020	54 (Initial Virus Spread)	Zhou et al. (2020) [45]
	24 January 2020–24 March 2020	60 (Global Spread and Market Collapse)	Kraemer et al. (2020) [46]
	25 March 2020–1 May 2020	36 (Policy Intervention)	Hale et al. (2021) [47]

## Data Availability

The original contributions presented in this study are included in the article. Further inquiries can be directed to the corresponding author(s).

## Appendix A

**Table A1:** Stock Index and Abbreviations for Emerging Market Countries.

Country	Stock Index	Abbreviation
China	Shanghai Securities Composite Index	SHCOMP
India	BSE SENSEX 30	SENSEX
Brazil	Índice Bovespa	IBOVESPA
Russia	Russian Trading System	RTS
South Africa	FTSE/JSE Africa Top40 Tradeable Index	FTSE/JSE Top 40 Index
Indonesia	Indonesia Jakarta Composite Index	JKSE
Argentina	S&P Merval	MERV
Mexico	S&P/BMV IPC Index	MXX
Turkey	ISE National-100 index	XU100
Saudi Arabia	Tadawul All Share Index	TASI

**Table A2:** Results of the Robustness Test: Adjustments of the Transfer Entropy Function Parameters.

Results of the Parameter of the ETE Function: shuffles = 50, bootstrap = 300										
	China	India	Brazil	Russia	SouthAfrica	Indonesia	Argentina	Mexico	Turkey	SaudiArabia
China	0.0000	0.0062	0.0043	0.0040	0.0036	0.0014	0.0000	0.0023	0.0026	0.0004
India	0.0022	0.0000	0.0039	0.0046	0.0039	0.0047	0.0018	0.0034	0.0030	0.0013
Brazil	0.0039	0.0167	0.0000	0.0129	0.0165	0.0152	0.0024	0.0014	0.0076	0.0048
Russia	0.0029	0.0035	0.0051	0.0000	0.0032	0.0065	0.0021	0.0036	0.0011	0.0032
SouthAfrica	0.0041	0.0106	0.0049	0.0005	0.0000	0.0051	0.0022	0.0061	0.0013	0.0013
Indonesia	0.0030	0.0060	0.0047	0.0035	0.0054	0.0000	0.0019	0.0037	0.0034	0.0009
Argentina	0.0044	0.0056	0.0023	0.0029	0.0042	0.0084	0.0000	0.0003	0.0028	0.0012
Mexico	0.0059	0.0166	0.0018	0.0081	0.0137	0.0167	0.0024	0.0000	0.0066	0.0038
Turkey	0.0039	0.0036	0.0036	0.0002	0.0018	0.0066	0.0013	0.0037	0.0000	0.0024
SaudiArabia	0.0018	0.0047	0.0072	0.0047	0.0049	0.0031	0.0003	0.0092	0.0039	0.0000
Results of the Parameter of the ETE Function: shuffles = 100, bootstrap = 100										
	China	India	Brazil	Russia	SouthAfrica	Indonesia	Argentina	Mexico	Turkey	SaudiArabia
China	0.0000	0.0062	0.0044	0.0040	0.0036	0.0012	0.0000	0.0022	0.0025	0.0004
India	0.0023	0.0000	0.0039	0.0046	0.0038	0.0047	0.0018	0.0034	0.0031	0.0014
Brazil	0.0038	0.0166	0.0000	0.0129	0.0166	0.0152	0.0024	0.0014	0.0077	0.0049
Russia	0.0030	0.0036	0.0051	0.0000	0.0032	0.0064	0.0020	0.0036	0.0012	0.0030
SouthAfrica	0.0039	0.0106	0.0051	0.0007	0.0000	0.0050	0.0022	0.0062	0.0014	0.0013
Indonesia	0.0030	0.0061	0.0049	0.0035	0.0054	0.0000	0.0020	0.0038	0.0035	0.0010
Argentina	0.0043	0.0056	0.0023	0.0029	0.0043	0.0085	0.0000	0.0003	0.0028	0.0011
Mexico	0.0059	0.0167	0.0017	0.0080	0.0137	0.0169	0.0026	0.0000	0.0066	0.0038
Turkey	0.0041	0.0037	0.0036	0.0003	0.0019	0.0067	0.0015	0.0037	0.0000	0.0024
SaudiArabia	0.0018	0.0048	0.0070	0.0047	0.0048	0.0033	0.0002	0.0091	0.0038	0.0000
